# Prognostic alternative splicing events related splicing factors define the tumor microenvironment and pharmacogenomic landscape in lung adenocarcinoma

**DOI:** 10.18632/aging.204244

**Published:** 2022-08-24

**Authors:** Jichang Liu, Yadong Wang, Xiaogang Zhao, Kai Wang, Chao Wang, Jiajun Du

**Affiliations:** 1 Institute of Oncology, Shandong Provincial Hospital, Shandong University, Jinan, Shandong, P.R. China; 2 Department of Thoracic Surgery, Shandong Provincial Hospital, Shandong University, Jinan, Shandong, P.R. China; 3 Department of Thoracic Surgery, The Second Hospital of Shandong University, Jinan, Shandong, P.R. China; 4 Department of Respiratory, The First Affiliated Hospital of Shandong First Medical University, Jinan, Shandong, P.R. China

**Keywords:** LUAD, alternative splicing, splicing factors, tumor microenvironment, immunotherapy

## Abstract

Background: Recent studies identified correlations between splicing factors (SFs) and tumor progression and therapy. However, the potential roles of SFs in immune regulation and the tumor microenvironment (TME) remain unknown.

Methods: We used UpSet plots to screen for prognostic-related alternative splicing (AS) events. We evaluated SF patterns in specific immune landscapes. Single sample gene set enrichment analysis (ssGSEA) algorithms were used to quantify relative infiltration levels in immune cell subsets. Principal component analysis (PCA) algorithm-based SFscore were used to evaluate SF patterns in individual tumors with an immune response.

Results: From prognosis-related AS events, 16 prognosis-related SFs were selected to construct three SF patterns. Further TME analyses showed these patterns were highly consistent with immune-inflamed, immune-excluded, and immune-desert landscapes. Based on SFscore constructed using differentially expressed genes (DEGs) between SF patterns, patients were classified into two immune-subtypes associated with differential pharmacogenomic landscapes and cell features. A low SFscore was associated with high immune cell infiltration, high tumor mutation burden (TMB), and elevated expression of immune check points (ICPs), indicating a better immune response.

Conclusions: SFs are significantly associated with TME remodeling. Evaluating different SF patterns enhances our understanding of the TME and improves effective immunotherapy strategies.

## INTRODUCTION

Alternative splicing (AS) is a crucial process in most gene expression and generates more than one mRNA from a single gene locus to then generate distinct proteins [1, 2]. More than 90% of human genes undergo AS, with variability between tissues [3]. Studies have shown that AS defects significantly affect cell development and underlie many diseases, including different cancers, as selective AS allows cancer cells to generate isoforms that benefit cell survival [4, 5]. Also, AS affects tumorigenesis by influencing genome instability [6] and gene mutations, other cancer hallmarks [7]. Splicing factor (SF) dysregulation is the main cause of aberrant AS, with SFs functioning as both oncoproteins and tumor suppressors [8].

Globally, lung cancer has the highest cancer related mortality [9]. Non-small cell lung cancer (NSCLC) accounts for the majority of lung cancers, of which lung adenocarcinoma (LUAD) ratios are continuously increasing [10]. Altered AS occurs in LUAD and mainly results from SF expression, including SRSF and RBM families [3, 11]. It was reported that aberrant AS events in LUAD contribute to different cell functions, such as apoptosis, proliferation, and drug resistance [11]. Although the mechanisms underpinning aberrant AS patterns remain unclear, assessing such patterns to evaluate LUAD risk and predict the effects of LUAD treatment may improve patient survival.

In recent years, LUAD treatments have been continuously developed and improved, but a definitive cure remains elusive [12]. Chemotherapy is the first line of treatment for lung cancer; however, its benefits have plateaued. Treatments targeting epidermal growth factor receptor (EGFR) mutations, anaplastic lymphoma kinase (ALK) fusion oncogenes, and *KRAS* mutations are considered promising but their effects are limited [13].

The tumor microenvironment (TME) plays a vital role in tumor proliferation, invasion, and migration [14]. Consistent with these functions, immunotherapy, including the anti-tumor effects of CTLA-4 and programmed death-ligand 1/programmed death-1 (PD-L1/PD-1) blockade, have become leading and powerful treatments for LUAD treatment [15–17]. TME complexity is mainly reflected by immune infiltration, and is based on the spatial localization of immune cells relative to the tumor and stromal compartments, therefore human tumors are categorized as immune-inflamed, immune-desert, and immune-excluded phenotypes, and present many challenges for tumor immunotherapy [15, 18].

Studies have identified relationships between AS or SFs and the immune microenvironment, including head and neck squamous cell carcinoma, breast cancer, and LUAD [19, 20]. It was reported that *PD-L1* generated a long non-coding RNA by AS to promote LUAD progression [21]. Recent studies also reported interactions between SFs and cancer therapy that some SFs contributed to drug resistance [13, 22]. In terms of cancer immunotherapy, peptides may serve as neoepitopes from tumor specific mRNA splicing events, with a potential to bind to major histocompatibility complex class I molecules [23]. Furthermore, splicing-derived neoantigens may be useful as predictive response biomarkers for immune check point (ICP) blockade therapies, such as PD-1 or CTLA-4 [23]. There is an urgent need to identify SF related targets for cancer therapy, and recognizing the significance of SFs and TME characteristics, mediated by SFs, will improve our understanding of tumor immunity.

Thanks to the establishment of public databases and bioinformatics, the analysis of tumor expression profiles is both rapid and convenient. In this study, we downloaded gene expression data from The Cancer Genome Atlas (TCGA), Gene Expression Omnibus (GEO), and TCGA SpliceSeq databases. By analyzing SF patterns and SF cluster-related differentially expressed genes (DEGs), we constructed several TME models to predict immunotherapeutic benefits in LUAD. We showed that AS is key for shaping the TME and may have important therapeutic applications for LUAD.

## METHODS

### Data sources and preprocessing

AS data from LUAD patients were downloaded from the TCGA SpliceSeq database, and included seven different AS events: 1) alternate acceptor site (AA), 2) alternate donor site (AD), 3) alternate promoter (AP), 4) alternate terminator (AT), 5) exon skip (ES), 6) mutually exclusive exons (ME), and 7) retained intron (RI). Specimens were included for further analysis if percentage percent-spliced-in (PSI) value were > 75%. An SF list was identified from a published study [24]. Gene expression and clinical data were respectively retrieved from TCGA (https://portal.gdc.cancer.gov/) and GEO (https://www.ncbi.nlm.nih.gov/geo/) databases. In total, six eligible LUAD cohorts (GSE13213 [25], GSE37745 [26], GSE31210 [27], GSE3141 [28], GSE30219 [29], and GSE50081 [30]) were integrated as the training cohort. LUAD patients from TCGA were the testing cohort. Batch effects from non-biological technical biases were corrected using the “ComBat” algorithm of the sva package. Copy number variation (CNV) data from TCGA-LUAD patients were obtained from the UCSC Xena website (https://xena.ucsc.edu/).

### Prognosis-related AS events

We eliminated specimens without follow-up or short follow-up information (< 90 days) to exclude the impact of short-term follow-up. AS events were eliminated when the standard deviation of the PSI value among specimens was < 0.01. Prognosis-related AS events were identified using univariate Cox regression analysis, and displayed in a volcano plot and UpSet map. The top 20 AS events were displayed in a bubble chart.

### Constructing an AS-SF interaction network

Correlations between prognostic AS events and SFs were evaluated by Spearman’s analyses in LUAD patients from the TCGA dataset. Interactions were included when correlation coefficients were > 0.6 and *p* < 0.001. Then, an AS-SF interaction network was constructed and included prognostic AS events and related SFs.

### SF-based consensus molecular clustering

In our AS-SF interaction network, we identified 16 SFs, including *CIRBP, CCDC130, CLASRP, LUC7L3, CLK1, CLK4, ALYREF, RBM5, CDK10, SREK1, SNRNP70, RBM15, ARGLU1, SRSF5, SRRM2*, and *SRSF11*. Genes, which were highly correlated with prognosis-related AS events. Four genes not found in the training cohort (integrated GEO cohort) were eliminated. Unsupervised clustering analysis was used to identify distinct patterns using these SFs. A consensus clustering algorithm was used to classify LUAD samples into different SF modification clusters and test corresponding stability. The ConsensuClusterPlus package [31] was used to perform these steps and 1000 times repetitions were calculated to guarantee corresponding stability.

### Gene set variation analysis (GSVA)

To explore biological heterogeneity between different SF patterns, GSVA enrichment was performed in the “GSVA” package [32]. Hallmark gene sets “h.all.v7.4.symbols.gmt” were extracted from the MSigDB database [33] to conduct GSVA. The clusterProfiler R package [34] was used to perform functional annotations for SF-related genes. An adjusted *P* value < 0.05 was considered statistically significant.

### Single sample gene set enrichment analysis (ssGSEA)

We used the ssGSEA algorithm to quantify the relative abundance of 28 infiltrating immune cell types, 13 immune functions, and other related biological processes in LUAD samples. The gene sets for marking each TME infiltration immune cell type stored various immune cell subtypes, including activated B cells, activated CD8 T cells, activated dendritic cells, macrophages, natural killer T cells, and regulatory T cells [35]. We extracted 13 gene sets for other related biological processes from published studies, including (1) immune checkpoints; (2) angiogenesis; (3) antigen processing machinery; (4) CD8 T effectors; (5) epithelial mesenchymal transition (EMT), including EMT1, EMT2, and EMT3; (6) pan-fibroblast TGFb response signatures (Pan-FTBRS); (7) Wnt targets; (8) mismatch repair; (9) DNA damage repair; (10) DNA replication; and (11) nucleotide excision repair [36, 37].

### Identifying DEGs between SF distinct clusters

We next determined SF-related DEGs among distinct SF patterns in LUAD. The limma package was used for this, and the filtering criteria for DEGs was an adjusted *P* value < 0.05.

### Constructing SFscore

An SF scoring approach was developed to quantify SF patterns from individual patients based on PCA values. DEGs selected from distinct SF phenotypes underwent prognostic analyses using univariate Cox regression model. Genes with a prognostic significance were extracted for next feature selection using recursive feature elimination in the ‘caret’ package. PCA analysis was then performed based on finally determined genes. Principal components 1 and 2 served as the signature score. Subsequently, a formula similar to previous studies was constructed to define the SFscore: SFscore = ∑(PC1_i_ + PC2_i_), where i = SF-related signature gene expression [38].

### Assessing TMB and predicting ICI therapy responses

Maftools package was used to visualize somatic mutations based on the Mutation Annotation Format file in the TCGA cohort [39]. The Tumor Immune Dysfunction and Exclusion (TIDE) algorithm in TCGA cohort [40], including dysfunction and exclusion of infiltrating cytotoxic T lymphocytes (CTLs), was used to assess immune evasion mechanisms in the TCGA cohort [41]. A high TIDE level indicated a low response to immune checkpoint inhibitor (ICI) therapy. The tumor neoantigen burden, including clonal and sub-clonal neoantigen burdens, was also used to assess immunotherapeutic efficacy in the TCGA cohort [35]. Differential expression analysis of ICP genes was performed in the training cohort.

### Prediction of drug sensitivity and small molecular drugs

The R package “pRRophetic” was used to calculate the half maximal inhibitory concentration (IC_50_) of chemotherapy drugs [42]. DEGs between groups with high and low SFscore were identified by *p* < 0.05 and |logFC|>1 in “limma”. SFscore-related small molecular drugs were identified in the Connectivity Map database (CMap; https://clue.io/) after uploading down- and up-regulated genes [43].

### Quantitative real-time PCR

Quantitative real-time PCR Total RNA of carcinoid tissues was extracted with Trizol reagent (A2A0209, Accurate Biotechnology, China). cDNA was amplified with reverse transcription kit (A2A1386) was provided by Accurate Biotechnology Co. (China). The sequences of primers are in Supplementary Table 1. Gene expression levels were assayed by qRT-PCR using the Roche LightCycler^®^ 480 system (Roche, Basel, Switzerland) with the SYBR Green system (A2A1436, Accurate Biotechnology).

### Statistical analyses

Statistical tests were performed in R-4.0.2 software, and a two-sided *P* value < 0.05 was considered statistically significant. Statistical significance for normally distributed variables was analyzed using Student’s *t*-tests, while non-normally distributed variables were estimated using the Wilcoxon rank-sum test. Kruskal-Wallis tests were used to compare more than two groups, as nonparametric and parametric methods, respectively. Kaplan-Meier survival analyses were conducted using the ‘Survminer’ package to explore associations between SF patterns and prognoses. Continuous variables were dichotomized for overall survival (OS) before the log-rank test using optimal cutoff values as determined by the “surv_cutpoint” function in the “Survminer” package. After this, that, LUAD samples were categorized into high and low SFscore subgroups.

### Availability of data and materials

The dataset supporting the conclusions of this article is available in the TCGA website (https://portal.gdc.cancer.gov/) and GEO database (https://www.ncbi.nlm.nih.gov/geo/).

## RESULTS

### Total and prognosis-related AS events

**Figure 1:**
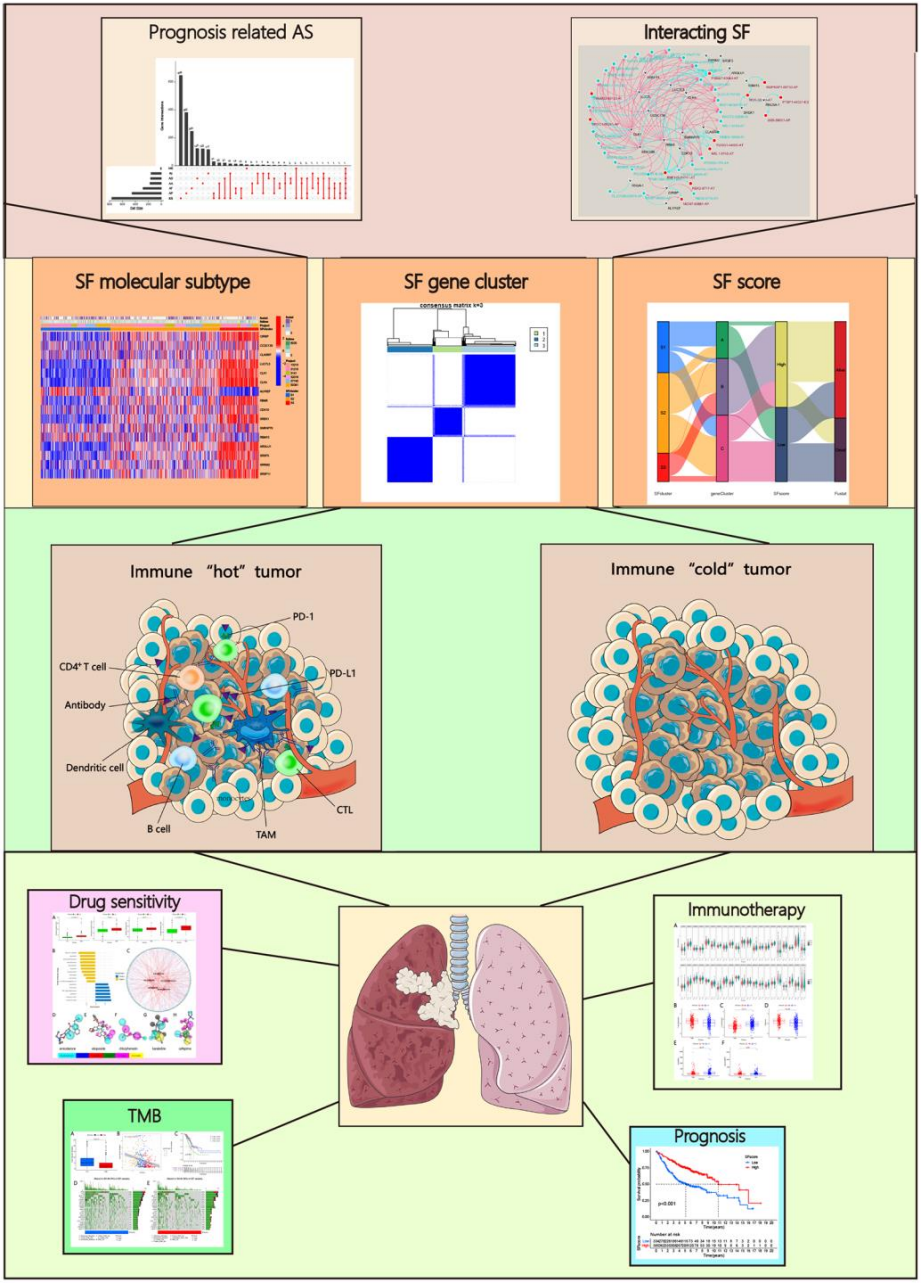
Analysis workflow of this study.

**Figure 2:**
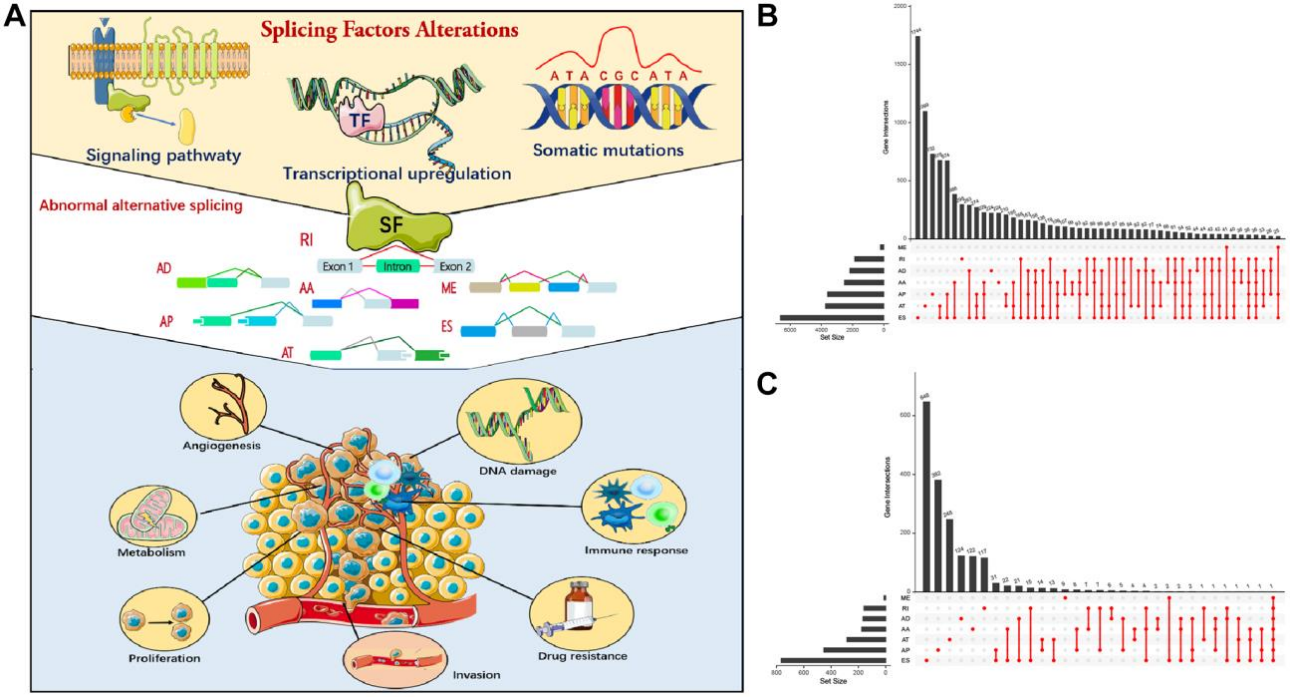
**Alternative splicing (AS) events in cancer.** (**A**) Mechanism of splicing regulatory factors in regulating RNA alternative splicing and tumor progression. ES (Exon skip) means that an exon is cut from the original transcript. RI (Retained intron): A new exon is formed by the retained Intron and the exons on both sides. AD (Alternate Donor site): The 3'-end splicing sites of different transcripts are the same but the 5'-end splicing sites are different. AA (Alternate acceptor site): The 5'-end splicing sites of different transcripts are the same but the 3'-end splicing sites are different. AP (Alternate promoter): The first exon of the two transcripts is different. AT (Alternate terminator): The last exon of the two transcripts is different. ME (Mutually exclusive exons): Different exons (called inclusive exons) are present in different transcripts. (**B**) Upset plot of all AS events in LUAD. (**C**) Upset plot of prognosis-related AS events in LUAD.

A flow chart (Figures 1, 2A) shows the study design and processes. In total, 572 LUAD specimens with 43,948 AS events in 10,366 gene symbols were collected. These events included 16,793 ES in 6,618 genes, 8,546 AT in 3,734 genes, 8,992 AP in 3,605 genes, 3,559 AA in 2,522 genes, 3,057 AD in 2,173 genes, 2,781 RI in 1,866 genes, and 220 ME in 214 genes. An UpSet plot was constructed to depict overlaps in the seven AS event types, which indicated multiple AS events appeared on a single gene (Figure 2B, 2C). ES was the most common ES event type, while ME was the least common.

Univariate Cox regression analysis was used to select prognosis-related AS events, but excluded patients with follow-up < 90 days. In total, 2,692 AS events significantly associated with OS (*p* < 0.05) were selected, including 906 ES, 487 AT, 744 AP, 185 AA, 182 AD, 177 RI, and 11 ME. A volcano map depicted AS events (Supplementary Figure 1A). The top 20 prognosis-related AS events of the seven types are shown (Supplementary Figure 1B–1H). Multivariate Cox analysis was also conducted to identify the independent prognostic factors (Table 1).

**Table 1:** Multivariate Cox analysis of all prognosis related AS events.

**Id**	**Coef**	**HR**	**HR.95L**	**HR.95H**	***p***
TTC39C|44852|AP	1.248442	3.484908	1.391101	8.730198	0.007711
CDKN2A|86004|AP	1.489648	4.435536	2.047892	9.60694	0.000158
C10orf32|12982|RI	−9.15774	0.000105	4.24E-07	0.026199	0.001137
BEST3|23330|AT	1.771023	5.87686	2.010846	17.1756	0.00121
TLE2|46644|AA	−25.1683	1.17E-11	4.38E-16	3.14E-07	1.31E-06
CA5B|98313|ES	−1.1536	0.315499	0.117144	0.849725	0.022483
SDCBP|83930|ES	−13.5109	1.36E-06	8.07E-10	0.002278	0.000363
HNRNPLL|53258|AT	−3.35378	0.034952	0.003746	0.326121	0.003247
MEGF6|315|ES	−1.42332	0.240914	0.095041	0.610679	0.002707

### Construction of an AS-SF interaction network

**Figure 3:**
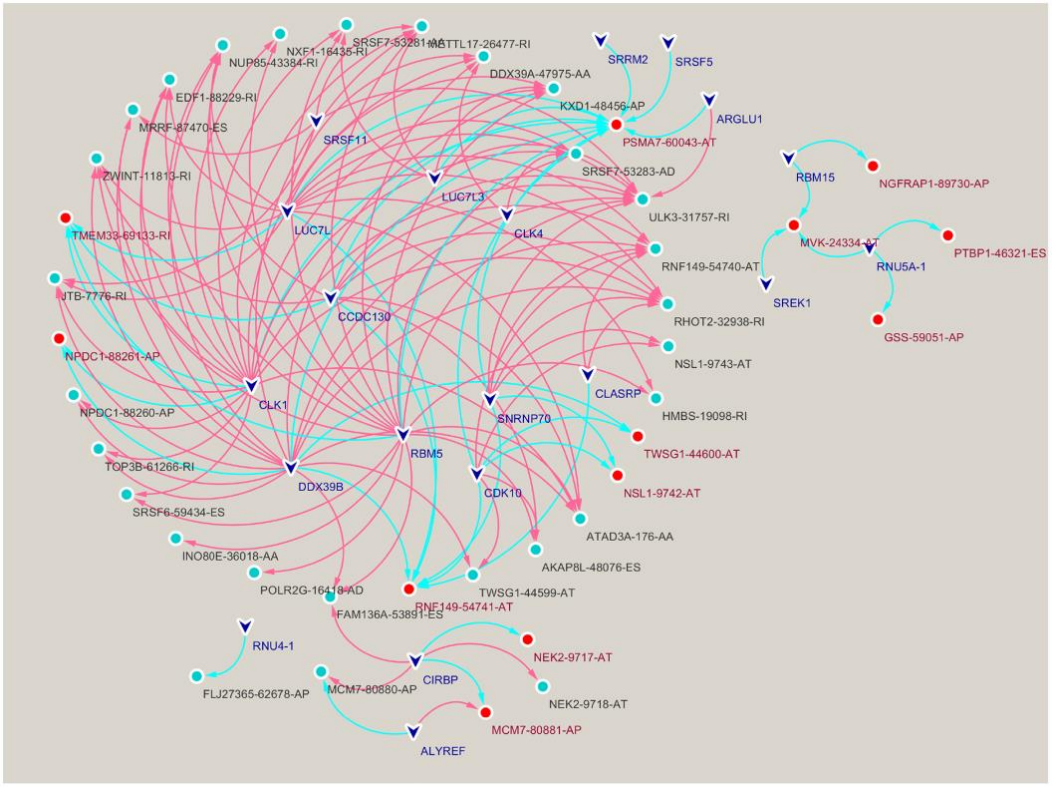
**Construction of a splicing factors (SFs)-alternative splicing (AS) events network.** Red edges mean positive regulation between AS and SF, while blue edges mean negative.

We performed correlation analysis between prognosis-related AS events and SFs, from which 20 prognosis-related SFs were selected (correlation coefficient > 0.6 and *p* < 0.001). A prognosis-related AS-SF interaction network was constructed, including 12 adverse AS events (red nodes), 28 favorable AS events (blue nodes), and 20 interacting SFs (black nodes) (Figure 3). Most adverse AS were negatively regulated by SFs, while MCM7-80881-AP was upregulated by ALYREF. In contrast, most favorable AS events were positively regulated by SFs, except MCM7-80880-AP and FLJ27365-62678-AP; they were downregulated by ALYREF and RNU4-1, respectively.

### The genetic variation of AS interacting SFs in LUAD

**Figure 4:**
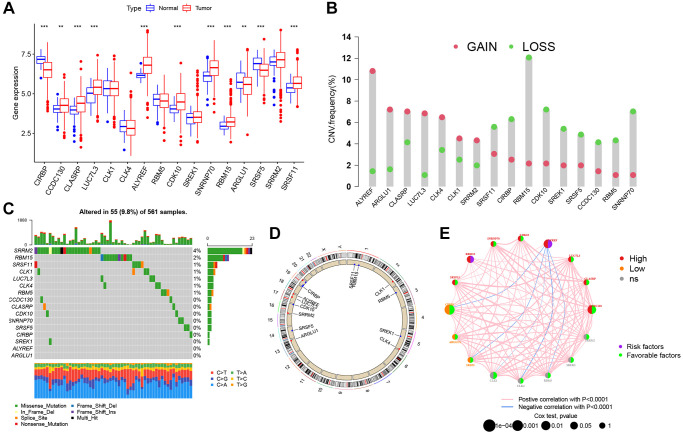
**Genetic alteration landscape of 16 splicing factors (SFs) in LUAD.** (**A**) Differential mRNA expression of 16 SFs between normal and tumor samples (^*^P < 0.05; ^**^P < 0.01; ^***^P < 0.001). (**B**) CNV mutation was widespread in 16 SFs. The column represented the alteration frequency. Deletion, green dot; Amplification, pink dot. (**C**) 56 of the 561 LUAD patients showed genetic alterations of 16 SFs. (**D**) The location of CNV alterations of 16 SFs on chromosomes. (**E**) The relationship between SFs in LUAD. The thickness of lines linking SFs showed the correlation strength. Negative correlation, blue; positive correlation, pink. Up-regulated SFs, red; down-regulated SFs, orange; no sense of SFs in differential expression, grey.

Based on the AS-SF interaction network, we identified 16 SFs (*CIRBP, CCDC130, CLASRP, LUC7L3, CLK1, CLK4, ALYREF, RBM5, CDK10, SREK1, SNRNP70, RBM15, ARGLU1, SRSF5, SRRM2*, and *SRSF11*) after excluding four genes (*LUC7L, DDX39B, RNU4-1*, and *RNU5A-1*) not found in training cohort. The differential expression of these SFs between normal and tumor specimens was analyzed in the TCGA cohort (Figure 4A). Most SFs were highly expressed in tumors, while *CIRBP, ARGLU1*, and *SRSF5* were poorly expressed. Also, 13 SFs were identified as significant prognostic risk factors in LUAD (Supplementary Figure 2). To understand the genetic variation landscape of SFs in LUAD, we explored the incidence of CNVs and somatic mutations in the 16 SFs. CNV alterations were widespread. *ALYREF, ARGLU1, CLASRP, LUC7L3, CLK4, CLK1*, and *SRRM2* were focused on the amplification in copy number, while the others had a frequency of CNV deletion (Figure 4B). Among the 561 LUAD samples, 55 had mutations in AS interacting SFs (Figure 4C). The chromosomal positions of CNV alteration in the 16 SFs are shown (Figure 4D). Based on the TCGA dataset, aa SF interaction network was constructed to show connections and the prognostic significance of SFs (Figure 4E). This network indicated that most SFs had positive correlation interactions, while *ALYREF* interacted with *CIRBP, SRSF5, CLK1, CLK4* and *RBM5* with negative correlations.

### Distinct SF patterns mediated by 16 AS interacting SFs

**Figure 5:**
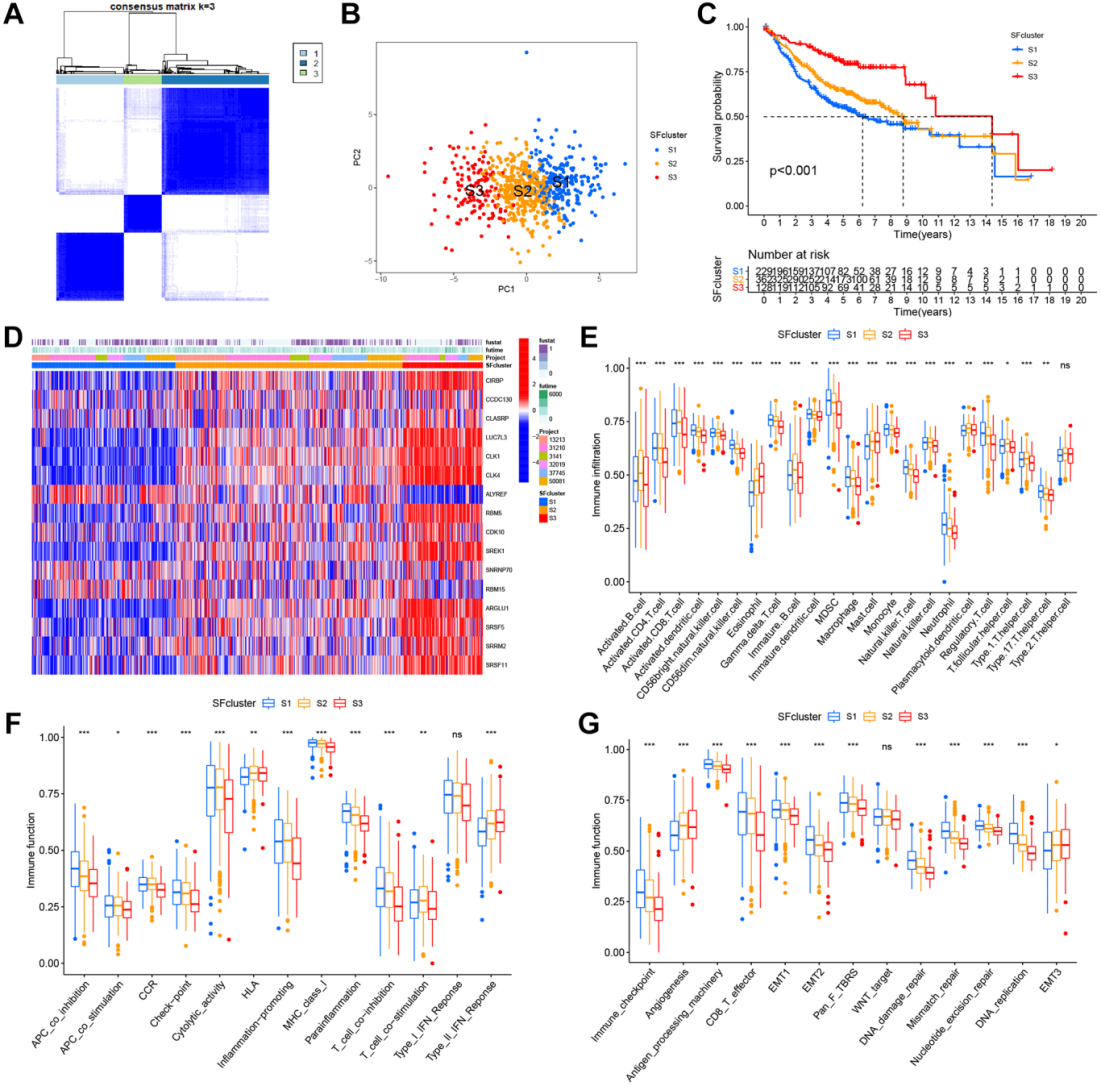
**SFs patterns and corresponding TME characteristics.** (**A**) Consensus clustering matrix for k = 3. (**B**) Principal component analysis (PCA) was conducted in distinct SFs patterns. (**C**) Survival analyses for three distinct SFs patterns based on six GEO cohorts (GSE13213, GSE37745, GSE31210, GSE3141, GSE30219 and GSE50081). (**D**) Heatmap of 16 SFs expression in LUAD patients. (**E**) TME cell infiltrating abundances in three SFs clusters. (**F**) Difference of immune functions in three SFs clusters. (**G**) Difference of other tumor-related biological processes.

Six GEO datasets (GSE13213, GSE37745, GSE31210, GSE3141, GSE30219, and GSE50081) were integrated into one meta-cohort. Based on SF expression, LUAD patients in the GEO meta-cohort were stratified into three distinct SF patterns using ConsensusClusterPlus in R (Figure 5A). We termed these patterns, SF clusters S1–S3, which included 229, 362, and 128 patients, respectively. PCA indicated significant distinctions in transcription profiles between patterns (Figure 5B). Survival analysis for these SF clusters showed a significantly prominent survival disadvantage in SF cluster-S1 (Figure 5C). Moreover, most SF genes were upregulated in SF cluster-S3 and downregulated in cluster-S1, while *ALYREF* and *RBM15* showed the opposite trend (Figure 5D, Supplementary Figure 3A).

### TME cell infiltration characteristics in distinct SF patterns

To explore biological function differences among the three SF patterns, we performed GSVA enrichment. As shown (Supplementary Figure 3B, 3C), when compared with SF cluster-S3, a series of carcinogenic and stromal pathways were activated in patients in SF-S1 and S2clusters, including MYC signaling, E2F signaling, mTROC1 signaling, and EMT. Additionally, immune-related pathways were fully activated in SF cluster-S2 patients, including interferon gamma/alpha responses, IL-6-JAK-STAT3 signaling, allograft rejection, and inflammatory responses. In contrast, SF cluster-S3 patients showed activated TGF-β signaling and suppressed immune functions.

In SF patterns, the ssGSEA algorithm was used to determine the abundance of different immune cell types and immune functions. Surprisingly, the patterns had significantly distinct TME cell-infiltrating and immune function characteristics (Figure 5E, 5F). Cluster-S1 was classified as an immune-inflamed phenotype, characterized by adaptive immune cell infiltration and immune function activation; cluster-S2 was identified as an immune-excluded phenotype, characterized by innate immune cell infiltration; and cluster-S3 was classified as an immune-desert phenotype with prominent immune suppression. Significant differences in TME cell-infiltrating composition and immune function were identified between clusters, and indicated these SFs had critical roles in TME remodeling. LUAD samples in cluster-S3 had a significantly lower abundance of all immune cells and immune functions, but a high abundance of eosinophils, mast cells, HLA, and type-II interferon responses. In contrast, clusters-S1 and S2 had a completely opposite landscape in terms of infiltrating cells and immune functions, therefore, cluster-S3 was classified as an immune “cold” phenotype, while Clusters S1 and S2 were immune “hot” phenotypes.

The three SF clusters also exhibited significant differences in distinct tumor-related functions from published research (Figure 5G). Cluster-S1 showed the highest level of most functions, excluding angiogenesis and EMT3, which were enriched in cluster-S3. In contrast, cluster-S3 had the lowest level of most functions, similar to above immune cells and functions, but angiogenesis and EMT3.

Specific correlations between TME infiltrating cell type and each SF were further examined using Spearman’s correlation analyses (Supplementary Figure 4). These SFs displayed mainly negative correlations with immune cell-infiltration abundance, except for *ALYREF* and *RBM15,* which were prominently and positively correlated with activated CD4+ T cells, gamma delta T cells, and CD56dim natural killer cells. Most SFs were related to the infiltration of T cells, including activated CD4+ T cells, gamma delta T cells, CD56dim natural killer cells, natural killer T cells, and regulatory T cells.

### SF cluster-related DEGs in LUAD

**Figure 6:**
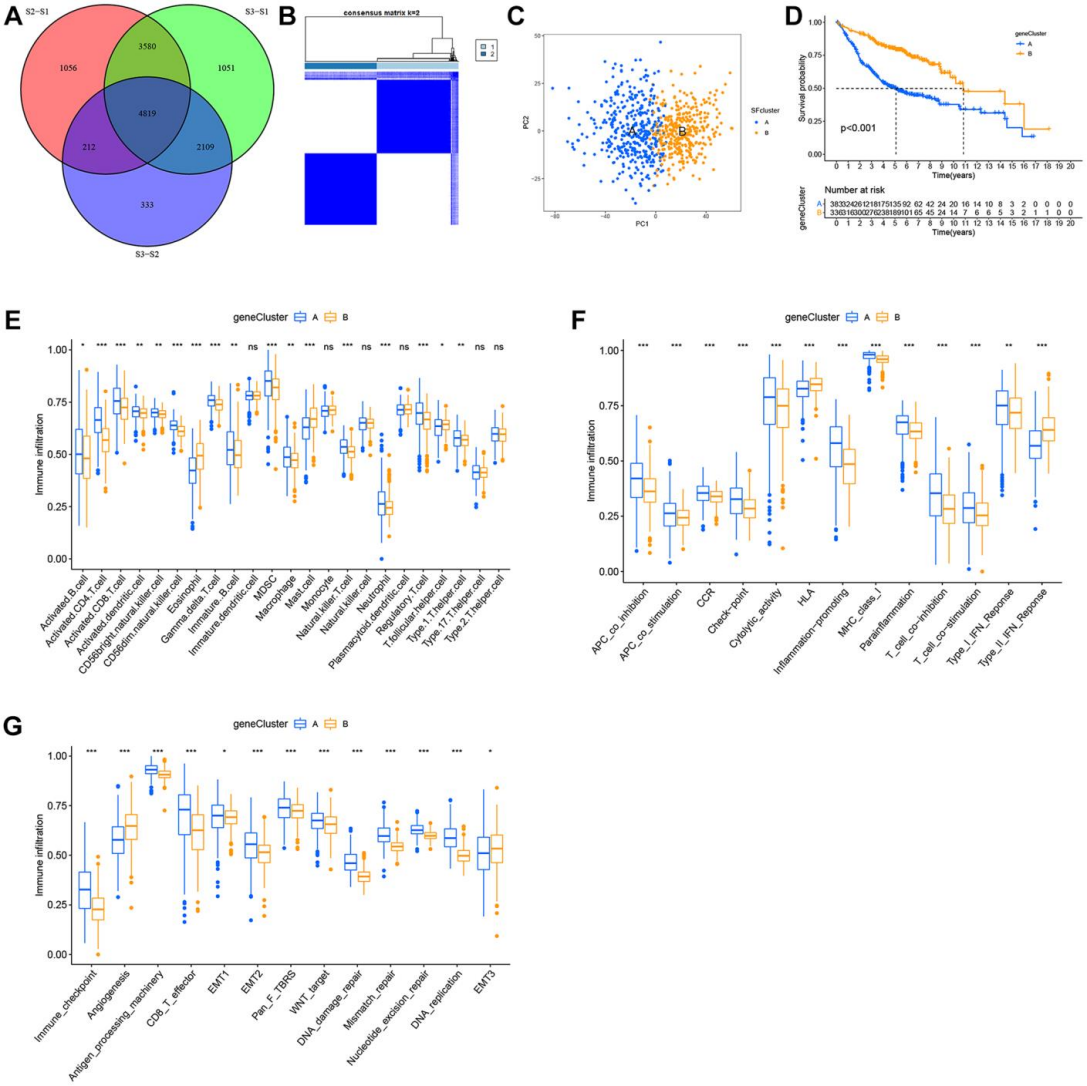
**Construction of SFs signatures.** (**A**) 4819 SFs-related differentially expressed genes (DEGs) between three distinct SFs patterns were presented in the Venn diagram. (**B**) Consensus clustering matrix for k = 3. (**C**) Principal component analysis (PCA) for the transcriptome profiles of gene cluster A, B. (**D**) Survival analysis for the two gene clusters based on 2959 SFs-related DEGs in GEO cohorts (*P* < 0.001). (**E**) TME cell infiltrating abundances in two gene clusters. (**F**) Difference of immune functions in two gene clusters. (**G**) Difference of other tumor-related biological processes.

Although LUAD patients were stratified into three SF patterns according to 16 SFs, the underlying genetic alterations were unknown. Therefore, we examined transcriptional changes among different SF patterns and identified 4,819 SF pattern-related DEGs in “limma” (Figure 6A). In total, 2,959 SF-related DEGs were screened out with prognosis significance based on Cox regression analyses. A gene expression heatmap is shown (Supplementary Figure 5A). We next performed unsupervised clustering analyses based on these 2,959 genes to identify different genomic subtypes. We then defined these three phenotypes as gene clusters A, B respectively (Figure 6B, 6C). Subsequent survival analyses showed a prominent prognosis advantage in cluster B (Figure 6D). We also examined the differential expression of the 16 SFs in these gene clusters (Supplementary Figure 5B); significant SF expression differences were observed - cluster B had a lower abundance of all immune cell types and immune functions, with a relevant higher abundance of eosinophils, mast cells, HLA and type-II interferon responses, while cluster A showed the opposite. Therefore, clusters A and B were classified as immune “hot” and “cold” phenotypes, respectively.

### The role of SF clusters in TME immune landscape remodeling

Consistent with immune phenotypes in SF clusters S1–S3, SF gene clusters A–C were characterized as immune-inflamed, immune-desert, and immune-excluded landscapes, respectively (Figure 6E, 6F). SF gene cluster B was identified with a relatively lower immune function and infiltrating abundance of most cells, and could be classified as an immune-inhibition and immune-desert group. In contrast, cluster A was significantly associated with an immune-inflamed status. Cluster C was defined as an immune-excluded phenotype. Similarly, SF gene clusters A–C exhibited a consistent trend in distinct tumor-related functions (Figure 6G).

### Construction of the SFscore

**Figure 7:**
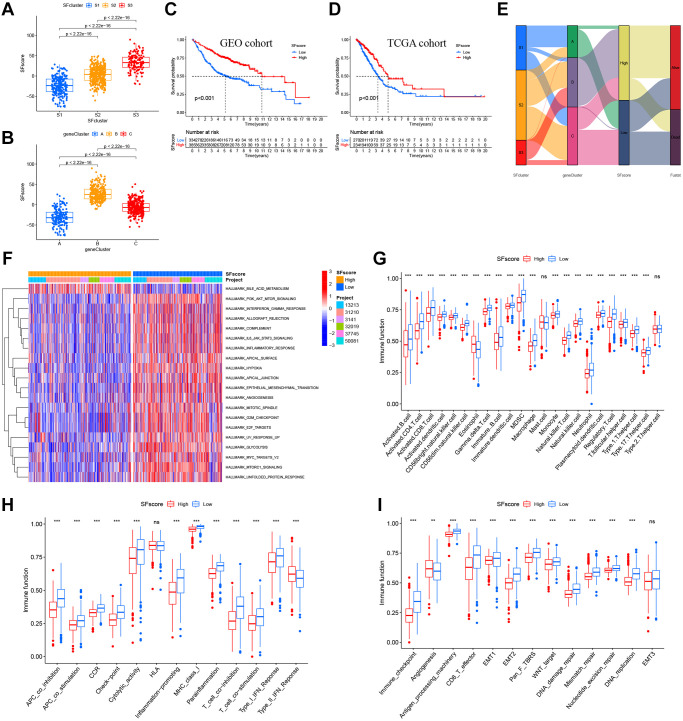
**Characteristics of SFscore in prognosis and TME landscapes.** (**A**) Differences in the SFscore between three SF clusters in LUAD (*P* < 2.22e-16). (**B**) Differences in the SFscore between two gene clusters in LUAD (*P* < 2.22e-16). (**C**) Kaplan-Meier curves for low and high SFscore patient groups in GEO cohorts (*P* < 0.001, Log-rank test). (**D**) Kaplan-Meier curves for low and high SFscore patient groups were validated in TCGA cohorts (*P* < 0.001, Log-rank test). (**E**) Alluvial diagram showing the changes of survival status, SFs clusters, gene clusters and SFscore. (**F**) GSVA enrichment analyses between groups with low/high SFscore. (**G**) TME cell infiltrating abundances in low/high SFscore groups. (**H**) Difference of immune functions in low/high SFscore groups. (**I**) Difference of other tumor-related biological processes in low/high SFscore groups.

Our results showed the non-negligible regulatory role of prognostic AS related SFs in shaping immune landscapes. Nevertheless, considering individual SF heterogeneity and complexity, a scoring system was required to accurately predict SF patterns in individual LUAD patients. Based on SF cluster-related DEGs, we developed SFscore to quantify SF patterns in patients. SF cluster-S3 and gene cluster B had the lowest SFscore (Figure 7A, 7B), thus, low scores were closely linked to immune-inhibition and immune-desert related clusters.

Subsequently, LUAD patients were divided into low or high SFscore groups using a cutoff value determined by the survminer package. A high SFscore was associated with a prominent survival benefit in GEO and TCGA cohorts (Figure 7C, 7D). Similarly, LUAD patients in SF cluster-S3 and gene cluster B had a better prognosis (Figure 7E). Moreover, we identified a prominent correlation between the SFscore and the risk score of a prognostic AS signature (Supplementary Figure 6A, 6B). Thus, a high SFscore was related to a low level AS risk score.

To further understand SFscore characteristics, we performed GSVA (Figure 7F) and showed that patients with high SFscore focused on the inhibition of immune pathways, carcinogenic pathways, and stromal pathways, including interferon gamma/alpha responses, IL-6-JAK-STAT3 signaling, allograft rejection, inflammatory responses, MYC signaling, E2F signaling, mTORC1 signaling, and EMT. Interestingly, bile acid metabolism was activated in the high SFscore group. We also examined correlations between TME cell infiltration abundance and SFscore (Supplementary Figure 6C). Interestingly, SFscore had significantly negative correlations with most immune infiltration cells, particularly activated CD4+ T cells, CD56dim natural killer cells, gamma delta T cells, MDSCs, natural killer T cells, and regulatory T cells (Supplementary Figure 6C). Similarly, ssGSEA showed a low abundance of most infiltrating immune cells and immune functions in groups with a high SFscore, except eosinophils and type-II interferon responses (Figure 7G, 7H). Also, a high SFscore was significantly related to lower tumor-related functions, including antigen processes, CD8 effectors, EMT, pan-F-TBRS, Wnt targets, DNA replication, DDR, mismatch repair, and nucleotide excision repair (Figure 7I). Nevertheless, angiogenesis was activated in groups with high SFscore. Finally, patients with high SFscore had low immune scores, stromal scores, ESTIMATE scores, and high tumor purity (Supplementary Figure 6D). In general, high SFscore were identified with immune-desert and stromal-inhibited landscapes, i.e., the immune “cold” group.

We also performed correlation analyses between SFscore and SF-related prognostic AS events in the network (Supplementary Figure 6A). Four AS events in two genes exhibited significant correlations with SFscore, including NEK2|9717|AT (cor = −0.65), NEK2|9718|AT (cor = 0.65), MCM7|80880|AP (cor = 0.63), and MCM7|80881|AP (cor = −0.63). Interestingly, the four AS events showed strong correlations with each other, and a distinct AS in a single gene showed an opposite correlation with the SFscore. Consistently, (Supplementary Figure 6B) these four AS events were significantly associated with multi-type immune cells, in particular activated CD4 T cells (cor > 0.5). Also, gamma delta T cells were widely associated with these selected AS events.

### Characteristic SFscore in tumor somatic mutations

**Figure 8:**
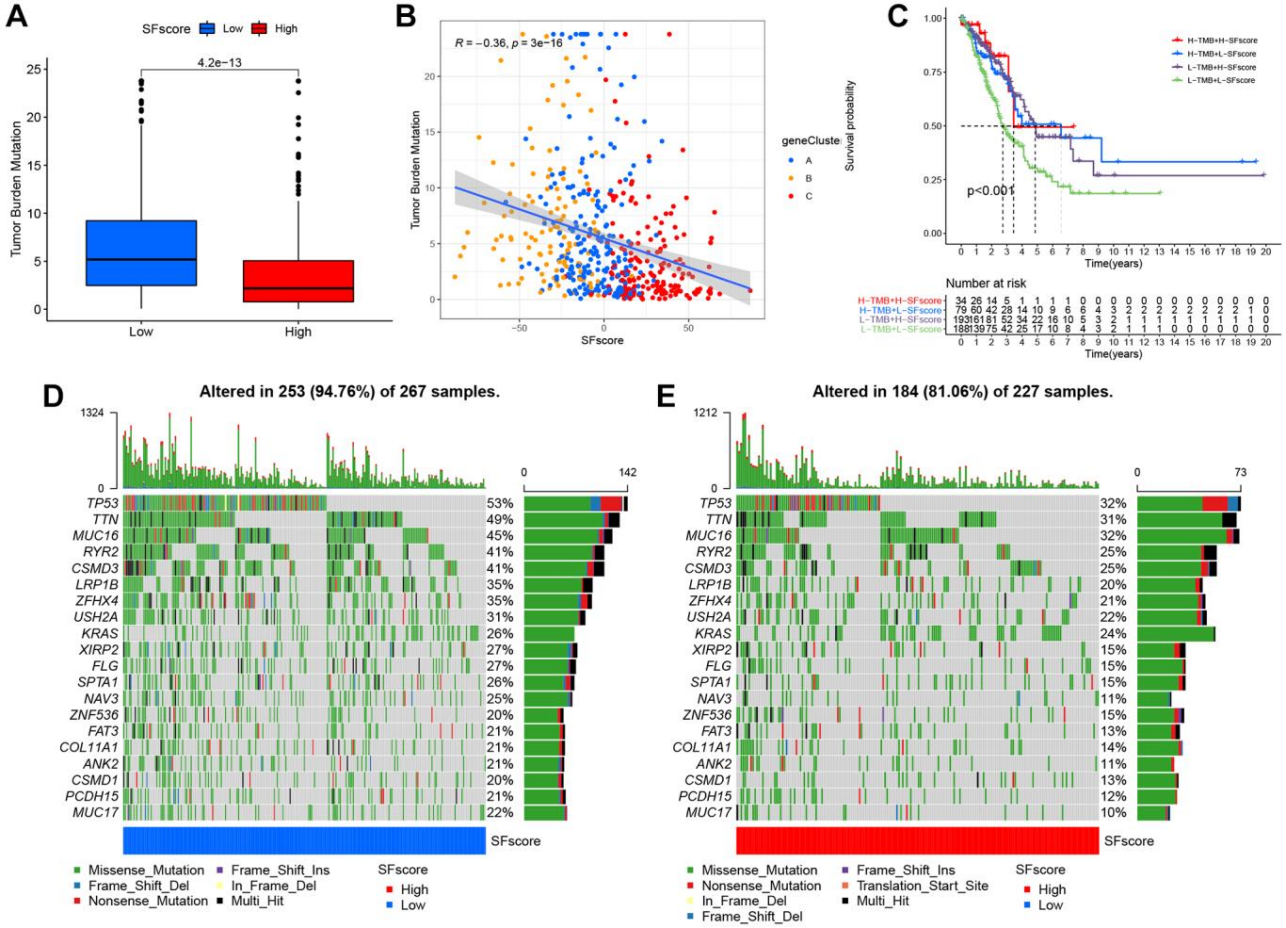
**Characteristics of SFscore in tumor somatic mutation.** (**A**) Differences in tumor mutation burden (TMB) between low and high SFscore groups of TCGA cohort (*P* < 0.001). (**B**) Correlations between SFscore and TMB using Spearman analysis (R = −0.36, *P* < 0.001). (**C**) Survival analyses for subgroup patients stratified by both SFscore and TMB using Kaplan-Meier curves. H-TMB, high TMB; L-TMB, low TMB (*P* < 0.001, Log-rank test). (**D**) The waterfall plot of tumor somatic mutation established by those with low SFscore. (**E**) The waterfall plot of tumor somatic mutation based on LUAD samples with high SFscore.

Several studies have highlighted associations between tumor somatic mutations and immunotherapeutic response [1, 44]. We explored the distribution patterns of tumor mutation burden in distinct SFscore groups and found that the low SFscore group had a higher TMB (Figure 8A). Additionally, the SFscore had a predominantly negative correlation with TMB across different gene clusters (Figure 8B). Survival analysis showed prominent differences among different TMB and SFscore combinations (Figure 8C). We next performed significantly mutated gene (SMG) analysis on LUAD samples in low SFscore versus high SFscore subgroups. LUAD samples with a low SFscore had significantly higher SMG rates (Figure 8D, 8E) and provided novel insights on SFscore in tumor somatic mutations, TME remodeling, and ICI therapy.

### The role of SFscore in predicting immunotherapeutic benefits

**Figure 9:**
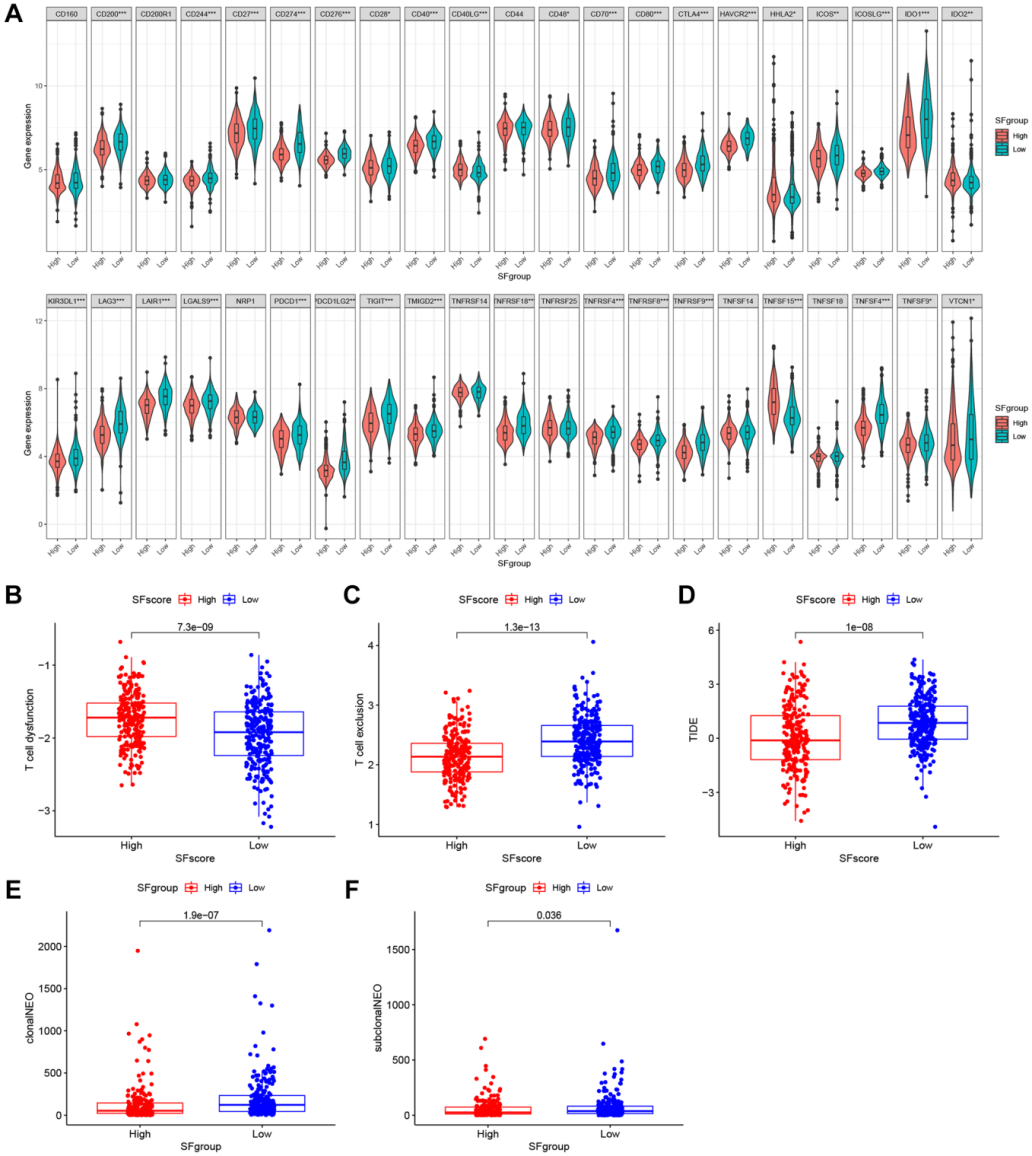
**The SFscore predicts immunotherapeutic benefits.** (**A**) The relative mRNA expression level of 42 immune checkpoints was compared between SFscore high versus low groups. (**B**–**D**) The relative distribution of T cell exclusion score, T cell dysfunction score and TIDE score were compared between SFscore high versus low groups in TCGA-LUAD cohort (*P* < 0.001). (**E**, **F**) The relative distribution of clonal neoantigens and sub-clonal neoantigens were compared between SFscore high versus low groups in TCGA-LUAD cohort (*P* < 0.05).

Immunotherapies, represented by ICP blockade, have emerged as major breakthroughs in cancer therapy. Currently, immunotherapy is considered a first-line treatment for patients with LUAD. We extracted 42 ICPs, including PD-1, PD-L1, and CTLA-4; the mRNA expression levels of most ICPs from low SFscore subtypes were significantly higher than those in high SFscore subtypes (Figure 9A). In addition to well-known ICPs, newly identified predictors, such as TIDE, are widely used and strongly recommended to evaluate immune response. We showed (Figure 9B–9D) that low SFscore patients were distinguished by a high level of T cell exclusion scores, TIDE and low T cell dysfunction scores. Tumor neoantigen burden, which is closely related to immunotherapeutic efficacy, was also assessed; LUAD patients with a low SFscore had a higher clonal and sub-clonal neoantigen burden (Figure 9E, 9F). The distinct distribution of these markers indicated that low SFscore patients could benefit from immunotherapy, especially ICIs. Thus, a crucial role for SFscore in mediating immune responses in patients was identified.

### Drug sensitivity analysis and small molecule drug screening based on SFscore

**Figure 10:**
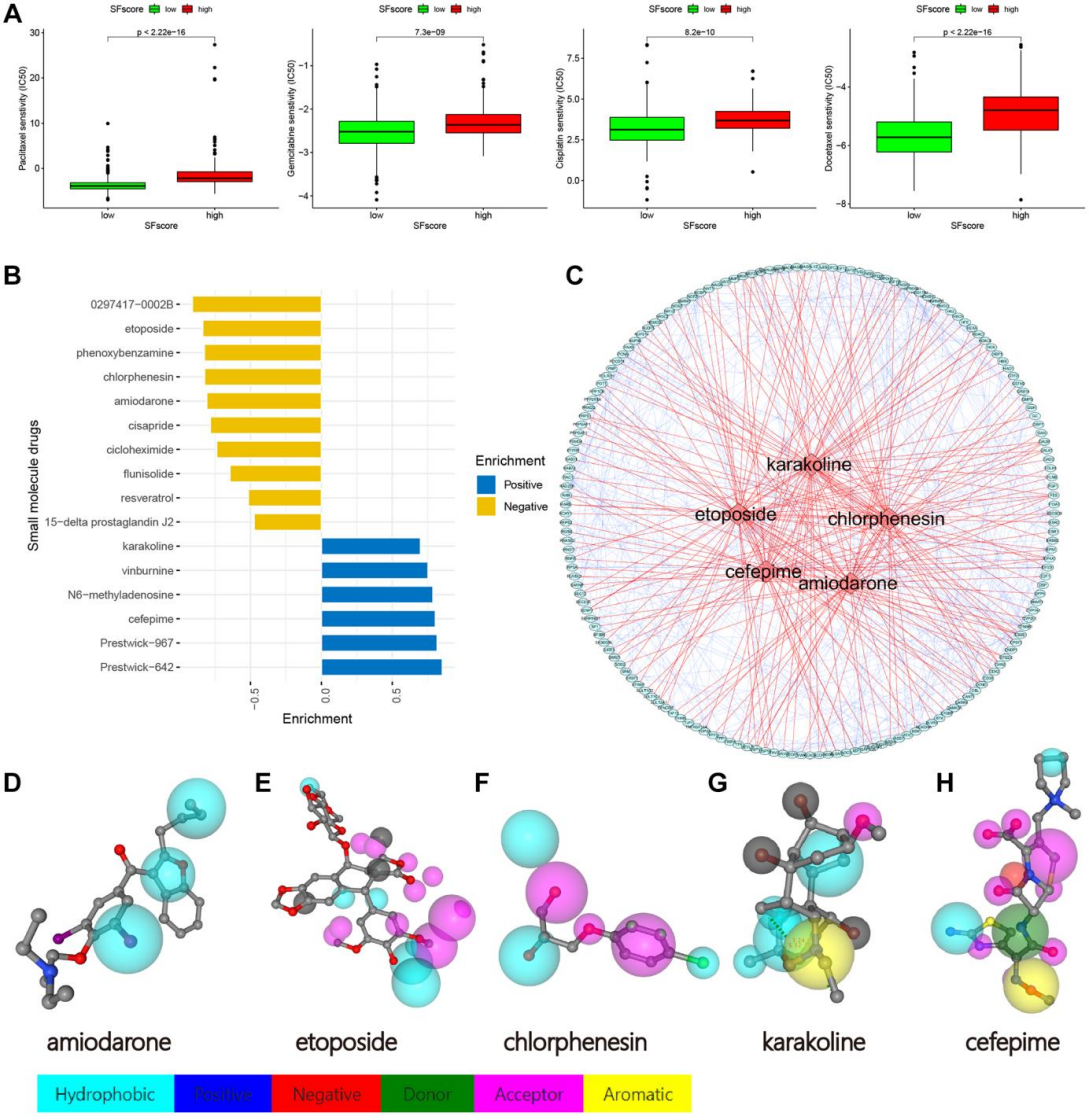
**Drug sensitivity analysis and small molecule drugs screening.** (**A**) Low SFscore is more sensitive to sorafenib, paclitaxel, sunitinib, cisplatin, docetaxel, Etoposide, vinorelbine, gemcitabine, gefitinib and vorinostat (*p* < 0.001). (**B**) Screening of small molecule drugs based on SFscore. (**C**) Network construction of target protein and five small molecule drugs. (**D**–**H**) Druggable pharmacophore models of five small molecule drugs.

We identified four chemotherapy drugs and estimated IC_50_ levels. As shown (Figure 10A), all four exhibited significantly low IC_50_ levels in the low SFscore group, including paclitaxel (*p* < 2.22e-16), cisplatin (*p* < 8.2e-10), docetaxel (*p* < 2.22e-16), and gemcitabine (*p* < 7.3e-09). Thus, low SFscore patients were more sensitive to the ten selected drugs. To further screen for specific drugs for patients with distinct SFscore, we analyzed DEGs between groups with distinct SFscore, including 111 up- and 106 down-regulated genes. DEGs were uploaded to the CMap database, and 16 small molecule drugs were identified (*p* < 0.01, |mean|>0.4) (Figure 10B). Among these, ten drugs targeting lower SFscore patients were selected with negative enrichment and six small molecule drugs were selected for higher SFscore patients (with positive enrichment). Amiodarone (*p* = 0.00056, enrichment = −0.809), etoposide (*p* = 0.00123, enrichment = −0.837), chlorphenesin (*p* = 0.00185, enrichment = −0.824), karakoline (*p* = 0.00201, enrichment = −0.699), and cefepime (*p* = 0.00277, enrichment = 0.804) were identified, and a network constructed to identify drug interactions with target proteins (Figure 10C). Also, druggable pharmacophore models of these five aforementioned drugs were analyzed and visualized in PharmMapper (Figure 10D–10H).

## DISCUSSION

While the LUAD incidence in NSCLC is increasing, the treatment did not receive promising effect [9, 10]. TME has a vital role in LUAD tumorigenesis, progression, metastasis, and therapeutic responses [14]. In the lung TME, tumor-infiltrating stromal cells are reprogrammed by malignant cancer cells, which in turn contribute to carcinogenesis [14]. However, the precise mechanistic details remain elusive.

Practically all protein-coding genes undergo one or more AS processes, including ES, AT, AP, AA, AD, RI, and ME. These AS forms require the spliceosome, which includes small nuclear ribonucleoprotein molecules (snRNPs, U1, U2, U4/U6, and U5) and other proteins, to generate different protein isoforms [45, 46]. SFs bind to pre-mRNAs which activate AS processes, to ultimately regulate cell function [47, 48].In our study, we constructed an AS-SF interaction network to identify SFs associated with AS events, and summarize interactions between OS-related AS events and SFs. This network included 12 adverse AS events (red nodes), 28 favorable AS events (blue nodes), and 20 interacting SFs (black nodes) (Figure 3), which suggested these SFs may be promising therapeutic targets.

It was reported that RBPs include two families: serine/arginine-rich proteins and heterogeneous RNPs (hnRNPs) which promote exon inclusion and exon skipping, respectively [46, 49]. Also, recurrent somatic mutations in SF genes may also directly affect cancer progression. Patients carrying the *SRRM2* missense genetic variant exhibited mis-splicing in specific cassette exons, while the variant segregated with familial papillary thyroid carcinoma [8, 50]. *RBM5, RBM6*, and *RBM10* are commonly deleted, mutated, and/or under-expressed or overexpressed genes in many cancers [8, 44, 51]; however, they have different roles in *in vitro* colony formation assays, partially due to their antagonistic regulation of NUMB alternative splicing [51].

Our LUAD data were divided into three patterns based on the expression of 16 SFs (Figure 5A, 5B). These patterns showed significant differences in OS (Figure 5C) and oncogenic pathways (Figure 5A, 5B), and indicated the important regulatory role of SFs in cancer progression.

Importantly, we matched these patterns with three major immunophenotypes, immune-inflamed, immune-desert, and immune-excluded phenotypes, based on the abundance of different immune cell types and immune functions (Figure 5E, 5F). Clusters S1–3 were classified as immune-inflamed, immune-excluded, and immune-desert phenotypes, respectively. Among clusters, there were apparently differences whether TME cell infiltration compositions or immune functions, which indicated the vital role of SFs in TME remodeling.

Previous studies demonstrated that TME contexture, including tumor-infiltrating CD4+/CD8+ T cells, macrophage M1, natural killer cells, and inflammatory cytokines, played vital roles in tumor progression and immunotherapeutic efficacy [18, 52]. We also showed that S3 patterns were significantly related to lower tumor-infiltrating CD8+ T cells, macrophage while elevated tumor-infiltrating eosinophil, HLA and type-II interferon response, which supported the potential value on immunotherapy and tumor progression.

To explore the underlying mechanisms of TME remodeling as mediated by SFs, we filtered all DEGs between the three SF patterns and screened out 2,959 DEGs which were significantly associated with OS (Figure 6D, Supplementary Figure 5A). Based on these DEGs, a different genomic subtype was identified (Figure 6B, 6C). Two transcriptomic subtypes, based on SF pattern genes, were significantly associated with different survival outcomes and TME landscape. Interestingly, similar to SF patterns, SF cluster-S3 and gene cluster B, classified as immune-desert and immune “cold” phenotypes, respectively, demonstrated better prognoses. As shown (Figure 5G and Figure 6G), these clusters were associated with many cancer-related phenotypes, including EMT and Wnt targets. Also, we observed significant differences in immune characteristics between SF clusters; however, this did not mean no immune cell infiltration or immune-related cell factors were present (Figure 5E, 5F and Figure 6E, 6F). In fact, the three immune infiltration phenotypes, which represented the degree of immune cell infiltration in this study, was a relative description. Also, the cancer-immune cycle assumes that the best anti-cancer immune response depends on cooperation between immune cells, host factors, and tumor antigens, and that this cycle must be efficiently initiated and precisely maintained, otherwise, the cancer-immunity cycle is broken and replaced by inflammation-promoted tumor progression [53–55]. In our study, the inflammation potentially occurring in relative immune “hot” phenotypes also exerted part of the immunosuppressive function, such as the up-regulation of immunosuppressive cells MDSC, Treg, etc. This may cause dysregulated anti-tumor immunity and mediate malignant progression. To a certain extent, this explains why SF cluster-S3 and gene cluster B had a relatively better prognosis.

We also constructed a quantification system “SFscore” to define different SF patterns and evaluate therapeutic effects and outcomes for LUAD patients. Our analyses highlighted that the SFscore was a prognostic biomarker for LUAD. Consequently, the immune “cold” phenotype showed the highest SFscore indicating a relatively low risk, while patterns characterized by the immune “hot” phenotype showed lower SFscore but with higher risk. As shown (Supplementary Figure 6D), the group with the highest SFscore was accompanied by a lower ImmuneScore, StromalScore, ESTIMATE score, and higher TumorPurity, indicating a lower level of immune cell infiltration. Similar to the SF cluster-S3 in hallmark LUAD, most signaling was downregulated in the high SF score group, including IL6/JAK/STAT3 signaling and inflammatory responses, and this downregulation was consistent with immune-desert characteristics.

It was reported that eosinophils have antitumorigenic roles in many cancers, including breast, colorectal, esophageal, and gastric cancers [56]. Activated eosinophils inhibited prostate cancer cell growth *in vitro* by secreting interleukin-10 (IL-10) and IL-12, and increasing E-cadherin expression, which putatively suppressed metastatic seeding [57]. Also, eosinophil-mediated cytotoxicity was reported in several co-culture studies of mouse or human eosinophils grown with cancer cells, including hepatocellular carcinoma cells, fibrosarcoma, melanoma, and CRC cells [58–61]. Interestingly, several mediators were shown to augment eosinophil-mediated killing, including interferon-γ [56]. In our study, type-II interferon was elevated in SF cluster-S3 and gene cluster B, with high SFscore and low risk, and bound the interferon-γ receptor to activate cell signaling pathways (involving JAK/STAT signaling). Consistently, human and mouse *in vitro* eosinophil activation with interferon-γ (but not TLR ligands) potentiated the eosinophil-mediated killing of CRC cells. Other studies suggested that interferon-γ and interferon-γ-induced pathways functioned as key regulators of eosinophil antitumorigenic activities [62, 63]. A collective view of these data indicated that eosinophil and type-II interferon had vital roles in antitumorigenic processes and, to a certain extent, explained the better prognoses in the group with elevated eosinophil and type-II interferon levels [56].

It was reported that gamma delta T cells had important roles in the development and progression of lung cancer and local microbiota by activating lung-resident gamma delta T cells and promoting inflammation associated with LUAD [64, 65]. One interesting finding from our study (Supplementary Figure 6B) showed that gamma delta T cells were negatively associated with all selected AS events. In fact, some AS events, such as NEK2|9717|AT and MCM7|80881|AP [66], are the most common form of gene expression that can perform right functions, and in this way, we may conclude that gamma delta T cell is closely related to gene AS and its function maybe affected by the special form of gene splicing. Interferon-γ induces CD8+ T cells to antigen (Ag)-specific CTLs and CD4+ T cell differentiation [67, 68], however, continuous exposure to interferon-γ may induce T cell exhaustion and tumor progression [69] by inducing immune escape mediators, including PD-L1, STAT3, and IDO1 [70, 71].

Recent studies also identified associations between high TMB and clinical benefit in NSCLC, melanoma, and bladder cancer patients when treated with PD-1/PD-L1 inhibitors or CTLA4 blockade [1, 44]. We analyzed correlations between TMB and SFscore; a higher SFscore was associated with lower TMB (Figure 8A, 8B) and the TMB of almost all common mutated genes in lung cancer decreased in the high SFscore cohort (Figure 8D, 8E). The L-TMB+L-SFscore cohort had the worst prognosis (Figure 8C) and no significant differences were identified between H-TMB+H-SFscore and L-TMB+H-SFscore cohorts. Therefore, TMB may be effective at predicting survival benefit in groups with relatively high degrees of immune infiltration, but not in the immune-desert phenotype.

When compared with the low SFscore cohort, almost all ICPs were decreased in the high SFscore cohort, and accompanied by low T cell exclusion, higher T cell dysfunction, and lower TIDE scores (Figure 9). These data suggested that SFscore may provide critical guidelines for clinical immunotherapy.

Apart from immunotherapy, we also investigated correlations between SFscore and the effects of other common chemotherapy drugs; we observed significantly different effects between high and low SFscore cohorts (*p* < 0.001).

Importantly, we identified different tumor immune phenotypes based on different AS events, provided new insights for improving patient clinical responses to immunotherapy, and promoted personalized cancer immunotherapy in the future.

## CONCLUSIONS

We demonstrated some of the extensive regulatory mechanisms underpinning AS events in the TME. Difference in AS events and SF patterns between patients are factors that cannot ignored and may cause TME heterogeneity and complexity in individuals. A comprehensive evaluation of individual tumor AS events and SF patterns will enhance our understanding of TMEs and guide more effective therapeutic strategies for lung cancer.

## Supplementary Materials

Supplementary Figures

Supplementary Table 1
