# Supplementary material for: Prognostic alternative splicing events related splicing factors define the tumor microenvironment and pharmacogenomic landscape in lung adenocarcinoma
Source: Aging (Albany NY). 2022 Aug 24;14(16):6689–715. doi: 10.18632/aging.204244 (PMC9467413; doi:10.18632/aging.204244)
Supplement: Supplementary Figures [file aging-14-204244-s001.pdf]

SUPPLEMENTARY FIGURES

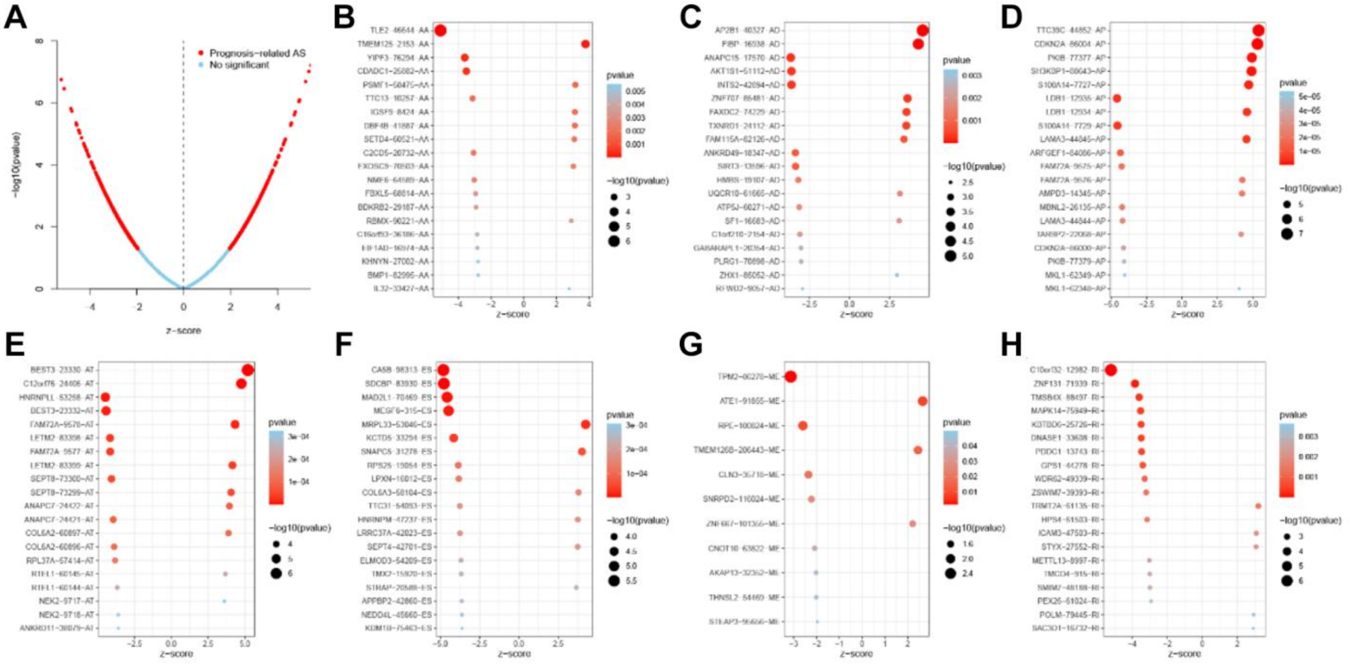

Supplementary Figure 1. Prognosis-related AS events in LUAD patients. (**A**) Prognosis-related AS events distributions in volcano plot. (**B**–**H**) Top 20 prognosis-related AS events of seven types in forest plot.

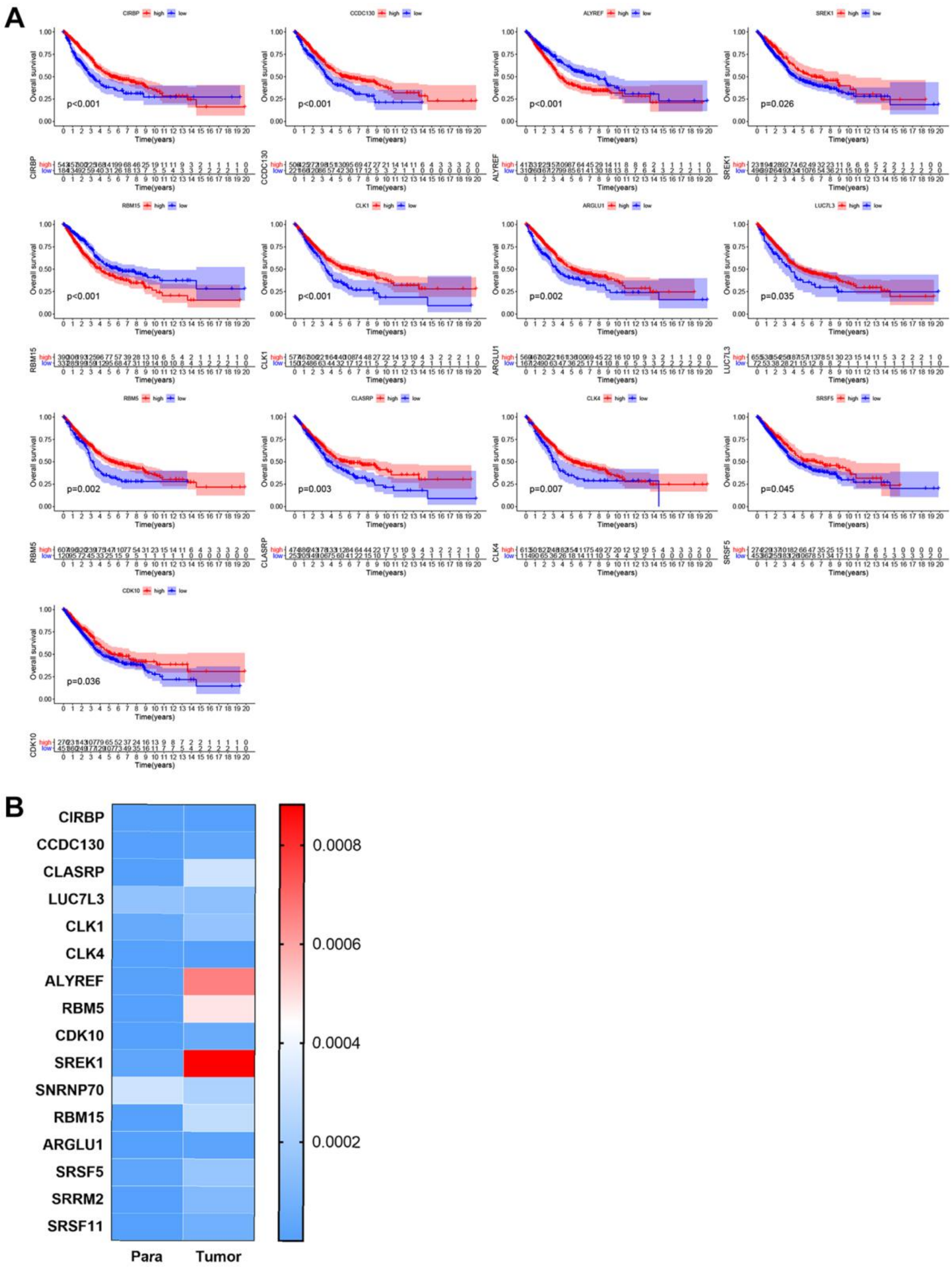

Supplementary Figure 2. (**A**) Kaplan-Meier survival analyses of SFs with prognostic significance; (**B**) expression level of SFs in surgical resected samples.

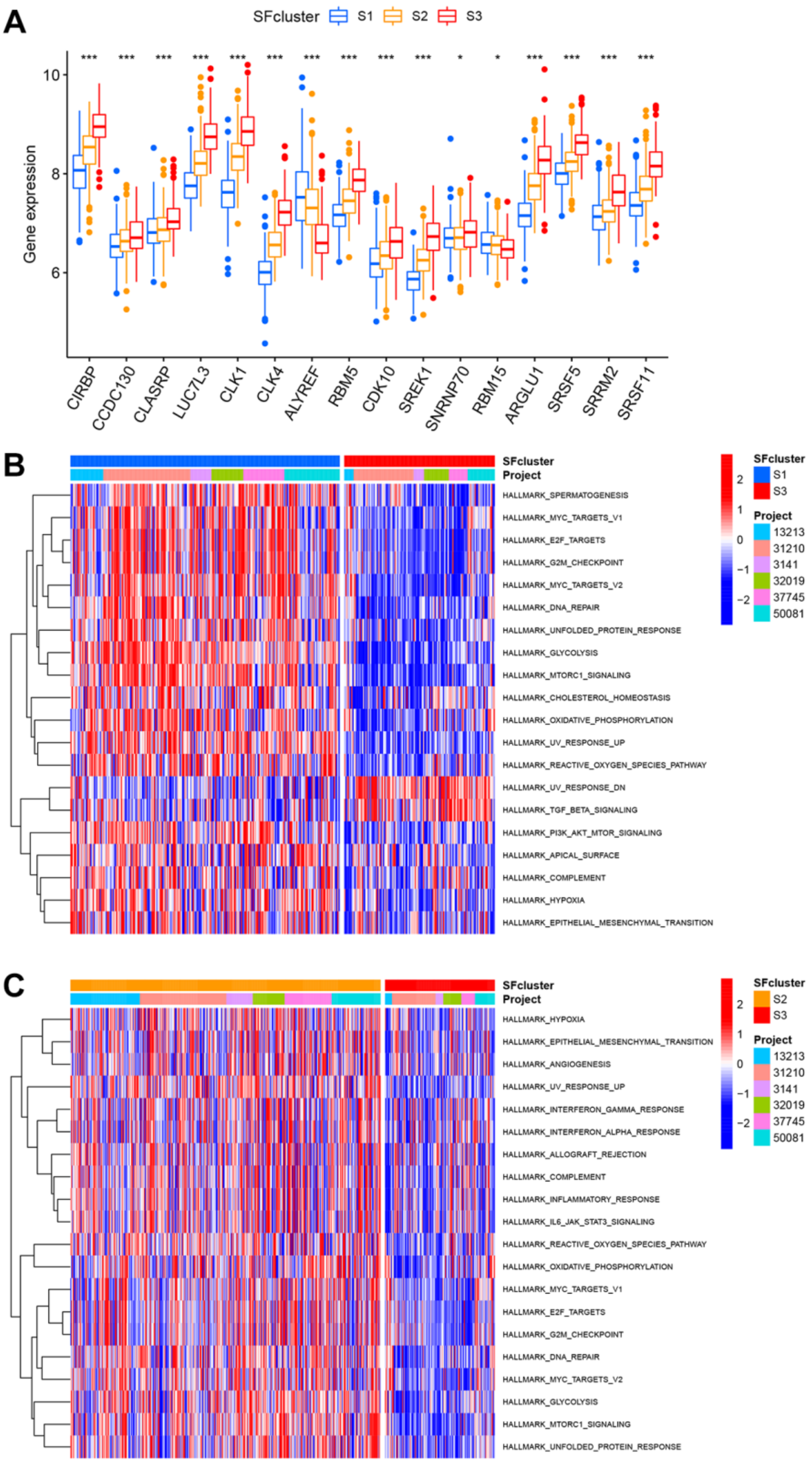

Supplementary Figure 3. (**A**) expression level of SFs in three SFs patterns; GSVA enrichment analyses based on the Hallmark gene set showed the states of biological processes of SFs cluster S1 vs. SFs cluster S3 (**B**) and SFs cluster S2 vs. SFs cluster S3 (**C**).

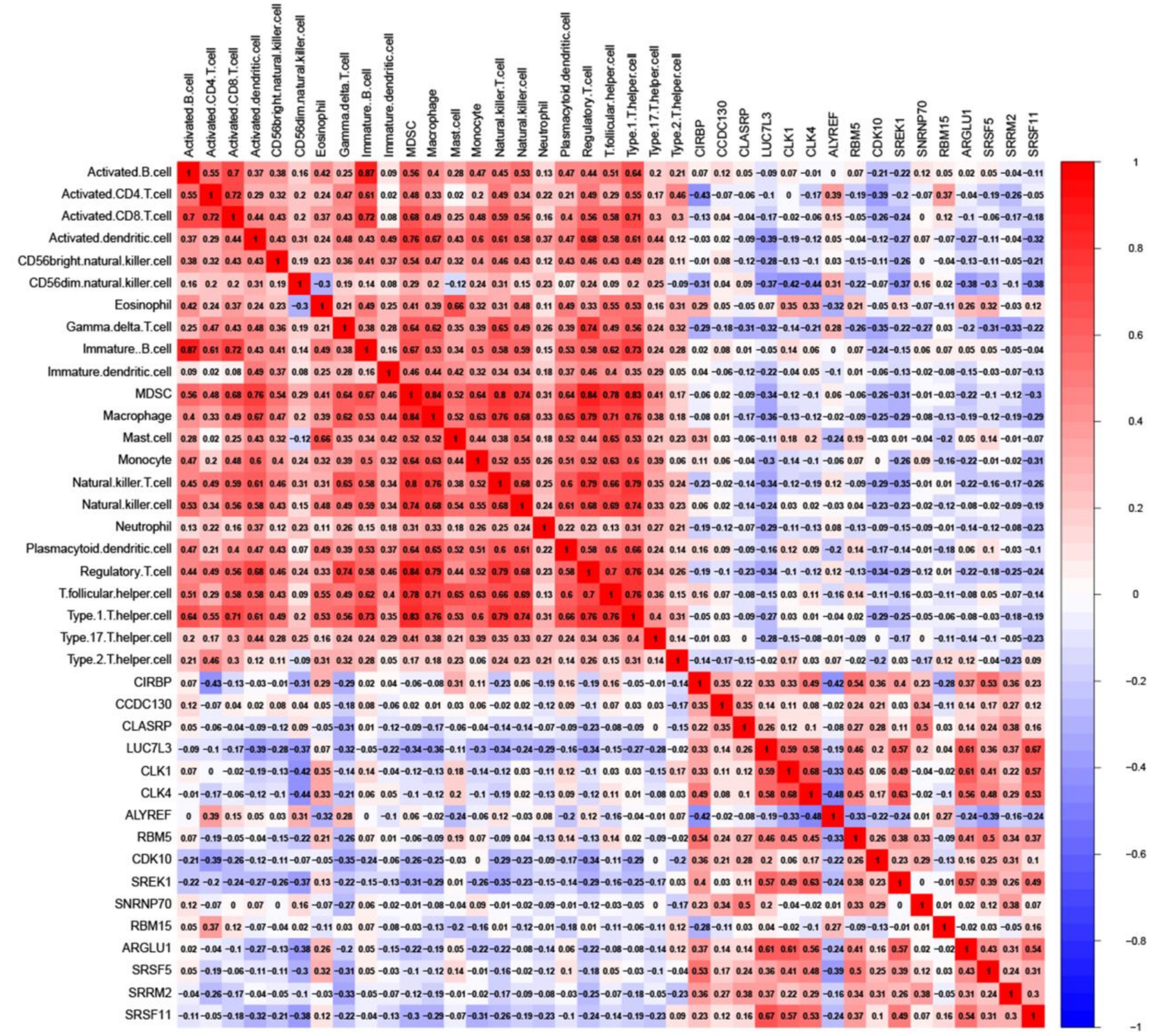

Supplementary Figure 4. Correlations between 16 SFs and 28 TME infiltrating cells in LUAD using spearman analysis.

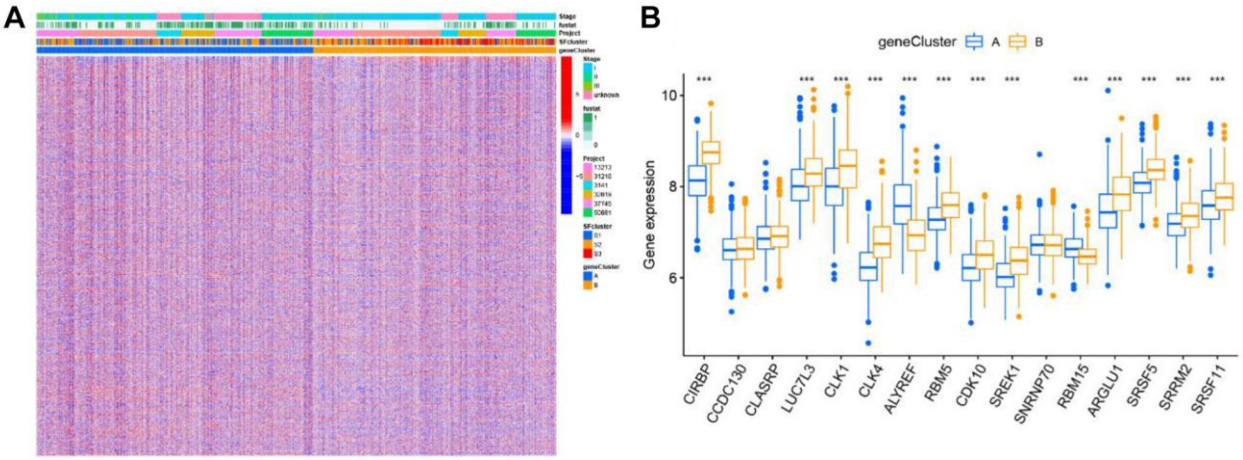

Supplementary Figure 5. (**A**) Unsupervised clustering of 2959 SFs -related DEGs with prognostic significance in GEO cohorts. (**B**) The expression level of SFs in two gene clusters.

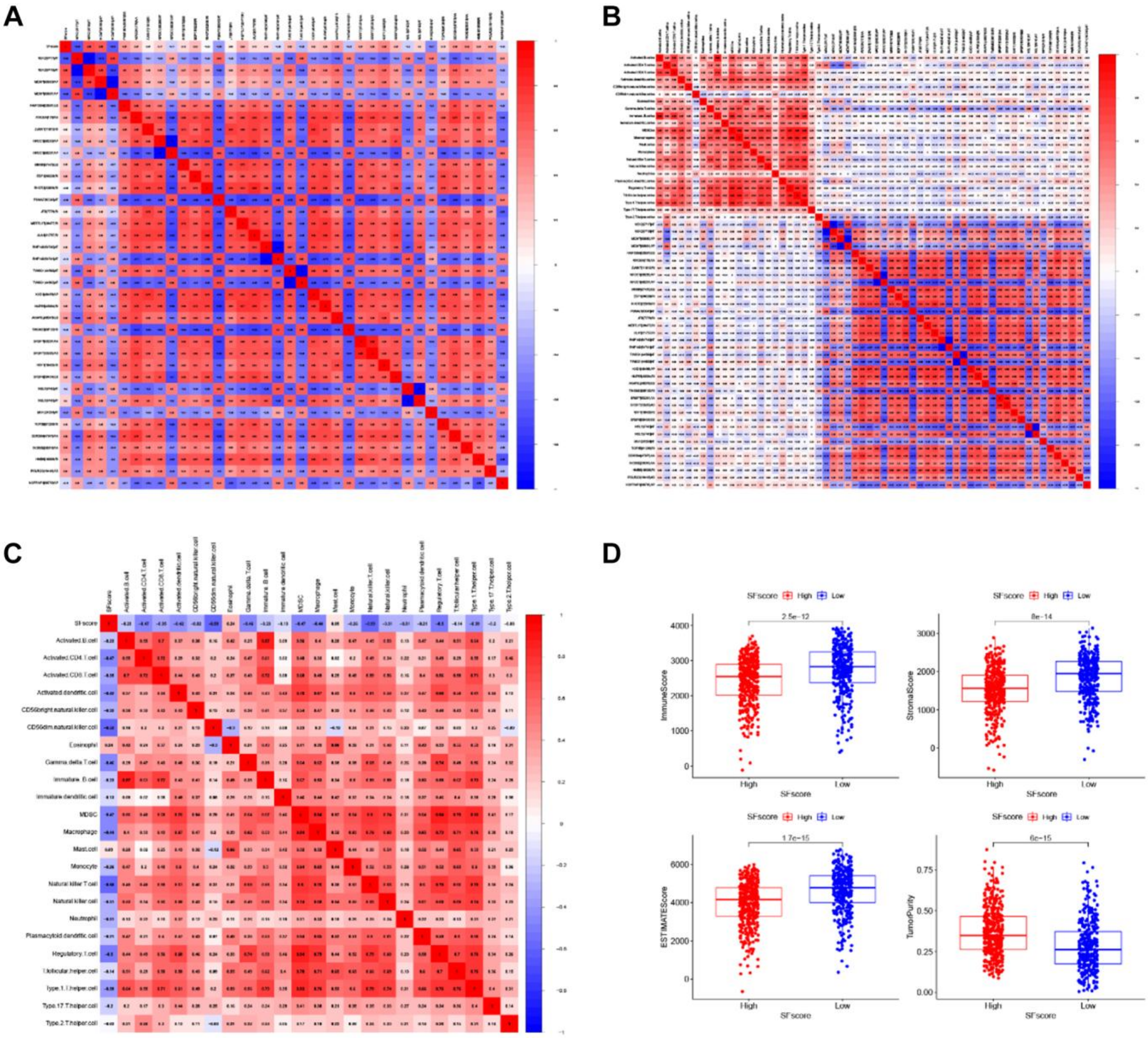

Supplementary Figure 6. (**A**) Correlations between SFscore and SFs interacting AS events. (**B**) Correlations between SFs interacting AS events and 28 TME infiltrating cells. (**C**) Correlation analysis between SFscore and TME infiltrating cells. (**D**) Difference of immune score, stromal score, ESTIMATE score and tumor purity in low/high SFscore groups.
