# Supplementary material for: Prognostic alternative splicing events related splicing factors define the tumor microenvironment and pharmacogenomic landscape in lung adenocarcinoma
Source: Aging (Albany NY). 2022 Aug 24;14(16):6689–715. doi: 10.18632/aging.204244 (PMC9467413; doi:10.18632/aging.204244)
Supplement: Supplementary Table 1 [file aging-14-204244-s002.pdf]

SUPPLEMENTARY TABLE

Supplementary Table 1. Sequences of primers.

Gene	Forward	Reverse
CIRBP	AGACTACTATAGCAGCCGGAGT	AAGGTGAACCGAGCTCCC
CCDC130	GCATCCTCATCATCCGATTCG	GTAGTAATTGCCCACCTTCTTCTT
CLASRP	TTTCATTTCCGCTTCCGGTG	ATGTTGTTGGTGTCCCCCTG
LUC7L3	TACTGTTTGACAGTGCAGCG	CTAATGGGGGCACGAGTCTG
CLK1	ATTTTGTTGTTGGTGCGCGA	TGAGTGTCTCATCGTCCTGG
CLK4	TGGCATGCATGTAGCAGTGA	GCATCTGGACACATCGGAAGA
ALYREF	AGAGCGTAAACAGAGGTGGC	ACTGGTGTCCATTCTCGCAT
RBM5	CCGGAGAGGACAGTGGATTG	TCAGACATGCTTGACCCACC
CDK10	ACGACCCTAAGAAAAGGGCG	GATCGGAAGACCTGGTGTGG
SREK1	GTCGTAGACGTTGGGGAGC	TCACCTGAATCACCGACGTG
SNRNP70	CGAGACATGCACTCCGCTTA	CTCTCATCGTAGCGGGAGGT
RBM15	ATCATTGTCCGTGGGTTTGGT	ACTATAACAGGGTCAGCGCC
ARGLU1	CGGCAGCGAAAAATTCGACA	CGCTCTAGCTCCTCACGTTT
SRSF5	GACCCCGTCCGGTAGGAAG	GACCGAGCCCTAGCATGTTC
SRRM2	CTCCGATACTTCCCGCAGTC	GTCGAGTTGCAGATTTCTCCT
SRSF11	CCTCTTCCCCCTCCTTCTCA	AAAGGCGAATCATCCGGCG
